# Bidirectional relationship between anxiety disorder and cancer: a longitudinal population-based cohort study

**DOI:** 10.1186/s12885-025-13930-6

**Published:** 2025-04-23

**Authors:** Sang-Hue Yen, Yi-Hsuan Hsu, Doreen Phiri, Chia-Chun Kuo, Hui-Fen Fang, Min-Huey Chung

**Affiliations:** 1 Division of Radiation Oncology, Department of Oncology, Taipei Veterans General Hospital, Taipei, Taiwan; 2 School of Nursing, Taipei Medical University, Taipei, Taiwan; 3 Ph.D. Program for Cancer Molecular Biology and Drug Discovery, College of Medical Science and Technology, Taipei Medical University and Academia Sinica, Taipei Medical University, Taipei, Taiwan; 4 Director of Administration Department, Taipei Cancer Center, Taipei Medical University, Taipei, Taiwan

**Keywords:** Anxiety disorder, Cancer, Longitudinal population-based study, Bidirectional

## Abstract

**Background:**

Although research has highlighted the link between anxiety and cancer, studies on the relationship between the two have produced inconsistent findings. Therefore, we investigated this relationship and also examined which types of cancer are more likely to induce anxiety.

**Methods:**

This retrospective longitudinal cohort study, conducted in Taiwan from 2003 to 2016, looked at the risk of cancer in 23,255 patients with anxiety disorder and the risk of anxiety in 33,334 patients with cancer diagnosed between 2003 and 2005. For both analyses, a comparison cohort was created using 1:4 case-control sampling. Cox proportional hazard regression models were used to analyze factors related to anxiety disorder or cancer.

**Results:**

Patients with anxiety were more likely to develop cancer (adjusted hazard ratio [AHR] = 1.29; 95% confidence interval [CI]: 1.23–1.35) compared to those in the comparison group. Particularly high risks were observed for thyroid cancer (AHR: 2.13, CI: 1.60–2.82), skin cancer (AHR: 2.10, CI: 1.63–2.71), and prostate cancer (AHR: 1.97, CI: 1.59–2.47). Patients with cancer were more likely to develop anxiety than those without cancer (AHR: 1.63, 95% CI: 1.56–1.71), with particularly high risks observed in those with nose cancer (AHR: 3.12, 95% CI: 2.41–4.03), leukemia (AHR: 2.54, 95% CI: 1.63–3.96), thyroid cancer (AHR: 2.34, 95% CI: 1.84–2.97), and oral cancer (AHR: 2.04, 95% CI: 1.65–2.52).

**Conclusions:**

Our findings highlight a bidirectional link between cancer and anxiety disorder. Understanding this two-way connection can help healthcare providers develop effective strategies for managing cancer and anxiety disorders.

## Introduction

Anxiety prevalence varies significantly, ranging from 1.1 to 66% in Asian populations [1] and 13.6-28.8% in Western populations [2]. Contributing factors include genetics, temperament, natural disasters, alcohol use disorders, and severe physical conditions like hemodialysis, chronic obstructive pulmonary disease and heart failure [3–9]. Anxiety has been linked to a deterioration in health status, poor prognosis, and low quality of life [10, 11]. Coronavirus disease 2019 (COVID-19) increased global anxiety cases by 25.6% [12] adding psychological and socioeconomic burdens [13]. Furthermore, anxiety has been associated with higher cancer risk, including urological and generalized anxiety disorder [14, 15], though some studies found no significant links. Thus, anxiety is a major public health concern with considerable personal health-related and socioeconomic implications [16].

Cancer is a leading cause of death, with 19.3 million new cases and 10 million deaths globally in 2020 [17, 18]. Anxiety in cancer patients ranges from 9.8 to 38% [19–21], and over half develop anxiety disorders post-diagnosis due to fear of cancer, poor understanding of the disease, treatment side effects, and fear of recurrence [22–25]. These factors can exacerbate conditions related to negative emotions, such as depression and anxiety, ultimately lowering patient quality of life and physical and mental well-being, as well as increasing mortality rates [26].

Research on anxiety and cancer has yielded mixed results [10] with some studies reporting higher anxiety risk in cancer patients, especially those with gynecological, lung, or hematological cancers, while others found no significant association [27]. Population-based studies have explored anxiety in cancer patients, but data on different cancer types in Taiwan remain limited [19, 21, 28, 29]. We therefore, investigated the bidirectional relationship between anxiety disorder and cancer in the Taiwanese population, assessing both the risk of cancer in those with anxiety and the risk of anxiety in those with cancer.

## Methods

### Data sources

We utilized the National Health Insurance Research Database (NHIRD), managed by the Health and Welfare Data Science Center, Ministry of Health and Welfare (HWDC, MOHW), which covers 96% of Taiwan’s population and 97% of its clinics and hospitals. The entire population file from NHIRD was used to analyze the risk of subsequent cancer. Medical claims records provided demographic information and clinical details, using ICD-9-CM codes (up to 2015) and ICD-10-CM codes (from 2016).

### Study sample

We conducted two analyses using the same participant selection process. Anxiety disorders were identified with ICD-9-CM codes 300.00-300.29, 309.21, 313.23, 293.84, and ICD-10-CM codes F41.9, F41.0, F41.1, F40.0x-F40.8, F93, F94.0, F06.4. Cancer was identified with ICD-9-CM codes 140–208 and ICD-10-CM codes C00-C96, D03. We included the 15 most common cancers in Taiwan: colon, lung, breast, liver, oral, prostate, thyroid, skin, stomach, gynecological, brain, urological, leukemia, bone, and nose cancer [30]. Breast and gynecological cancer analyses included only women, while prostate cancer analyses included only men.

### Procedure

In the first analysis, we examined the association between anxiety disorder and cancer risk. We selected patients with anxiety as their principal diagnosis from inpatient or outpatient claims between January 1, 2003, and December 31, 2005. The index date was the first anxiety-related visit, and patients with prior cancer were excluded. Patients were followed until cancer diagnosis, death, or December 31, 2016. The control group included patients without anxiety or cancer, matched 1:4 by age, sex, and index year.

In the second analysis, we investigated cancer’s association with anxiety disorder risk. Cancer patients were selected from claims data between January 1, 2003, and December 31, 2005, with the first cancer visit as the index date. Patients with prior anxiety were excluded. Follow-up continued until an anxiety diagnosis, death, or December 31, 2016. Controls without cancer or anxiety were matched 1:4 by age, sex, and index year.

Demographic data were retrieved from the NHIRD. Patients were grouped by age (< 30, 30–44, 45–64, ≥ 65 years), monthly income (< NT$20,000, NT$20,000-NT$39,999, ≥NT$40,000), and region (Northern, Central, Southern, Eastern Taiwan). Regions were further categorized as urban, suburban, or rural. Comorbidities were assessed using Charlson Comorbidity Index (CCI) scores [31], with patients classified as low (CCI < 3) or high (CCI ≥ 3) comorbidity.

### Statistical analysis

Statistical analyses were conducted using SAS version 9.4 (SAS Institute, Cary, NC, USA). The chi-square test compared demographic differences between groups, and incidence density rates were calculated by dividing incident cases by total person-years.

In the first analysis, a Cox proportional hazards model was used to calculate crude and adjusted hazard ratios (CHRs and AHRs) for cancer risk in individuals with anxiety, adjusting for age, sex, income, region, urbanization, and CCI score. A similar model was applied in the second analysis to assess anxiety disorder risk in cancer patients. Statistical significance was set at *p* <.05.

### Ethics approval and consent to participate

This study was conducted in accordance with the ethical guidelines outlined in the Declaration of Helsinki and was approved by the Taipei Medical University Joint Institutional Review Board (TMU-JIRB No. N202004116). The requirement for written informed consent was waived by the board due to the retrospective nature of the study.

## Results

### Anxiety and subsequent risk of cancer

In this analysis, 23,255 patients were in the anxiety group and 93,020 in the anxiety-free group (Table 1). Most anxiety patients were aged 45–64 (36.12%) or 30–44 (33.76%), were women (62.42%), had monthly incomes below NT$20,000 (62.37%), lived in Northern Taiwan (45.83%), and resided in urban areas (41.13%). The two groups differed significantly in income, region, and CCI scores, with those without anxiety having lower CCI scores.

**Table 1:** Demographic characteristics of patients with anxiety disorder and comparison cohort (2003–2005)

Variable	Patients with anxiety disorder (*N* = 23,255)		Patients without anxiety disorder (*N* = 93,020)		*p* value
	*N*	%	*N*	%	
**Age (years)**					
<30	3434	14.77	13,736	14.77	-
30–44	7850	33.76	31,400	33.76	
45–64	8400	36.12	33,600	36.12	
≥65	3571	15.36	14,284	15.36	
**Sex**					
Male	8739	37.58	34,956	37.58	-
Female	14,516	62.42	58,064	62.42	
**Income**					
≥NT$40,000	4038	17.36	13,726	14.76	< 0.0001
NT$20,000-NT$39,999	4713	20.27	18,356	19.73	
< NT$20,000	14,504	62.37	60,938	65.51	
**Region**					
Northern	10,658	45.83	45,240	48.63	< 0.0001
Central	3423	14.72	11,331	12.18	
Southern	8682	37.33	33,744	36.28	
Eastern	492	2.12	2705	2.91	
**Urbanization level**					
Urban	9564	41.13	38,309	41.18	0.2821
Suburban	4622	19.88	18,863	20.28	
Rural	9069	39	35,848	38.54	
**CCI**					
<3	13,000	55.90	72,038	77.44	< 0.0001
≥3	10,255	44.10	20,982	22.56	

CCI: Charlson comorbidity index

The cancer incidence rates in the patients with anxiety over the 14-year follow-up period are presented in Table 2. The cancer incidence was higher in those with anxiety disorder (1.08 vs. 0.90 per 100 person-years) than in those without anxiety disorder. Besides, in the higher CCIs group, all age groups, and both men and women, those with anxiety, and the lower CCIs group had a significantly higher incidence of cancer than those without anxiety disorder. Moreover, those with anxiety had a significantly higher risk of developing cancer than those without (CHR = 1.21; 95% CI: 1.16–1.26). After adjustment for age, sex, income, region, urbanization level, and CCI score, individuals with anxiety still had a higher risk of developing cancer than those without (AHR = 1.29; 95% CI: 1.23–1.35).

**Table 2:** Risk of cancer by demographics among individuals with anxiety disorder

Variable	Cancer				
	Cases	PY	Incidence	CHR (95% CI)	AHR (95% CI)
**All**					
Anxiety free	9572	1059057.9	0.90	ref	ref
Anxiety	2653	245448.99	1.08	1.21 (1.16–1.26)**	1.29 (1.23–1.35)**
**Age (years)**					
**< 30**					
Anxiety free	289	167366.51	0.17	ref	ref
Anxiety	102	39826.83	0.26	1.51 (1.20–1.89)**	1.41 (1.12–1.78)*
**30–44**					
Anxiety free	1918	375589.81	0.51	ref	ref
Anxiety	585	88077.34	0.66	1.32 (1.20–1.45)**	1.28 (1.16–1.41)**
**45–64**					
Anxiety free	4555	389938.09	1.17	ref	ref
Anxiety	1235	89075.67	1.39	1.20 (1.12–1.27)**	1.23 (1.15–1.31)**
**≥ 65**					
Anxiety free	2810	126163.49	2.23	ref	ref
Anxiety	731	28469.15	2.57	1.15 (1.06–1.25)**	1.31 (1.21–1.43)**
**Sex**					
**Female**					
Anxiety free	5600	683948.86	0.82	ref	ref
Anxiety	1572	155886.93	1.01	1.25 (1.18–1.32)**	1.34 (1.26–1.42)**
**Male**					
Anxiety free	3972	375109.04	1.06	ref	ref
Anxiety	1081	89562.06	1.21	1.14 (1.07–1.22)**	1.22 (1.14–1.31)**
**CCI**					
**< 3**					
Anxiety free	6752	829947.93	0.81	ref	ref
Anxiety	1391	138277.10	1.01	1.24 (1.17–1.31)**	1.41 (1.33–1.50)**
**≥ 3**					
Anxiety free	2820	229109.97	1.23	ref	ref
Anxiety	1262	107171.89	1.18	0.98 (0.92–1.05)	1.11 (1.04–1.19)*
**Urbanization level**					
**Urban**					
Anxiety free	3788	440498.31	0.86	ref	ref
Anxiety	1114	101344.07	1.10	1.29 (1.21–1.38)**	1.36 (1.27–1.45)**
**Suburban**					
Anxiety free	2022	211110.12	0.96	ref	ref
Anxiety	530	48352.11	1.10	1.16 (1.05–1.28)*	1.27 (1.15–1.40)**
**Rural**					
Anxiety free	3762	407449.47	0.92	ref	ref
Anxiety	1009	95752.81	1.05	1.15 (1.08–1.24)**	1.22 (1.14–1.31)**

Incidence: incidence density (100 per person-years)

*: *p* <.05, **: *p* <.001, CHR: crude hazard ratio, AHR: adjusted hazard ratio controlling for age, sex, income, region, urbanization level, and CCI score, CCI: Charlson comorbidity index, CI: confidence interval, PY: person-years

Table 3 presents the cancer incidence by cancer type as well as the CHR and AHR for patients with anxiety. The patients with anxiety exhibited particularly high risks of developing thyroid (AHR: 2.13, CI: 1.60–2.82), skin (AHR: 2.10, CI: 1.63–2.71), prostate (AHR: 1.97, CI: 1.59–2.47), nose (AHR: 1.84, CI: 1.39–2.44), brain (AHR: 1.78, CI: 1.26–2.51), urological (AHR: 1.40, CI: 1.16–1.69), breast (1.27, CI: 1.11–1.46), and lung cancers (AHR: 1.24, CI: 1.09–1.40).

**Table 3:** Risk of cancer by type among individuals with anxiety disorder

Variable	Patients with anxiety		Patients without anxiety		Hazard ratio and 95% CI	
	Cases	Incidence ^a^	Cases	Incidence ^a^	CHR (95 CI%)	AHR (95 CI%)
**Cancer type**						
Colon cancer	353	0.14	1458	0.14	1.06 (0.94–1.19)	1.18 (1.05–1.33)*
Lung cancer	324	0.13	1237	0.12	1.15 (1.01–1.29)*	1.24 (1.09–1.40)**
Breast cancer	284	0.18	1123	0.16	1.12 (0.98–1.28)	1.27 (1.11–1.46)**
Liver cancer	336	0.14	1072	0.10	1.36 (1.20–1.53)**	1.19 (1.05–1.35)*
Oral cancer	103	0.04	475	0.04	0.92 (0.74–1.13)	1.07 (0.86–1.34)
Prostate cancer	121	0.14	294	0.08	1.77 (1.43–2.19)**	1.97 (1.59–2.47)**
Thyroid cancer	75	0.03	165	0.02	2.02 (1.54–2.66)**	2.13 (1.60–2.82)**
Skin cancer	89	0.04	224	0.02	1.77 (1.38–2.27)**	2.10 (1.63–2.71)**
Stomach cancer	85	0.03	411	0.04	0.91 (0.72–1.15)	0.96 (0.76–1.22)
Gynecological cancer	148	0.09	707	0.10	0.91 (0.76–1.09)	1.01 (0.85–1.21)
Brain cancer	47	0.02	130	0.01	1.55 (1.11–2.17)*	1.78 (1.26–2.51)*
Urological cancer	150	0.06	471	0.04	1.40 (1.16–1.68)**	1.40 (1.16–1.69)**
Leukemia	41	0.02	150	0.01	1.20 (0.85–1.69)	1.24 (0.87–1.77)
Bone cancer	7	0.03^+^	24	0.02^+^	1.33 (0.57–3.10)	1.37 (0.57–3.27)
Nose cancer	74	0.03	181	0.02	1.71 (1.31–2.25)**	1.84 (1.39–2.44)**

^a^ Incidence: incidence density (100 per person-years)

^+^ Incidence: incidence density (1000 per person-years)

*: *p* <.05, **: *p* <.001, CHR: crude hazard ratio, AHR: adjusted hazard ratio controlling for age, sex, income, region, urbanization level, and CCI score

### Cancer and subsequent risk of anxiety

For this analysis, 33,334 and 133,336 patients were assigned to the cancer and cancer-free groups, respectively (Table 4). The patients with cancer predominantly were aged 45–64 or ≥ 65 years (42.10% and 34.92%, respectively), were men (50.59%), had a monthly income of < NT$20,000 (65.17%), lived in Northern Taiwan (45.19%), lived in urban areas (39.10%), and had high CCI scores (67.96%). The two patient groups differed significantly in income, region, urbanization level, and CCI score. In addition, the patients without cancer had significantly lower CCI scores than those with cancer.

**Table 4:** Demographic characteristics of patients with cancer and comparison cohort (2003–2005)

Variable	Patients with cancer (*N* = 33,334)		Patients without cancer (*N* = 133,336)		*p* value
	*N*	%	*N*	%	
**Age (years)**					-
<30	1236	3.70	4944	3.70	
30–44	6426	19.28	25,704	19.28	
45–64	14,033	42.10	56,132	42.10	
≥65	11,639	34.92	46,556	34.92	
**Sex**					-
Male	16,865	50.59	67,460	50.59	
Female	16,469	49.41	65,876	49.41	
**Income**					< 0.0001
≥NT$40,000	5453	16.36	19,396	14.55	
NT$20,000-NT$39,999	6156	18.47	22,081	16.56	
< NT$20,000	21,725	65.17	91,859	68.89	
**Region**					0.0002
Northern	15,062	45.19	61,135	45.85	
Central	4601	13.80	18,393	13.79	
Southern	12,724	38.17	49,559	37.17	
Eastern	947	2.84	4249	3.19	
**Urbanization**					< 0.0001
Urban	12,957	38.87	52,134	39.10	
Suburban	7085	21.25	29,620	22.21	
Rural	13,292	39.88	51,582	38.69	
**CCI**					< 0.0001
<3	10,679	32.04	75,454	56.59	
≥3	22,655	67.96	57,882	43.41	

CCI: Charlson comorbidity index

The incidence of anxiety was higher in the individuals with cancer (1.29 vs. 0.99 per 100 person-years) than in those without cancer (Table 5). In all age groups, both men and women and CCI groups, individuals with cancer had a significantly higher incidence of anxiety than those without cancer. Those with cancer had a higher risk of developing anxiety (CHR: 1.30, CI: 1.25–1.36) than did those without cancer. After adjustment for age, sex, income, region, urbanization level, and CCI score, the patients with cancer still had a higher risk of developing anxiety than those without cancer (AHR: 1.63, 95% CI: 1.56–1.71).

**Table 5:** Risk of anxiety disorder by demographics among individuals with cancer

Variable	Anxiety				
	Cases	PY	Incidence	CHR (95% CI)	AHR (95% CI)
**All**					
Cancer free	14,087	1420588.57	0.99	ref	ref
Cancer	2613	203262.40	1.29	1.30 (1.25–1.36)**	1.63 (1.56–1.71)**
**Age (years)**					
**< 30**					
Cancer free	297	57848.59	0.51	ref	ref
Cancer	101	11436.06	0.88	1.74 (1.39–2.18)**	1.81 (1.38–2.37)**
**30–44**					
Cancer free	2032	300535.67	0.68	ref	ref
Cancer	596	51080.13	1.17	1.75 (1.60–1.92)**	1.92 (1.73–2.14)**
**45–64**					
Cancer free	6887	648669.69	1.06	ref	ref
Cancer	1291	92827.35	1.39	1.32 (1.24–1.40)**	1.51 (1.42–1.61)**
**≥ 65**					
Cancer free	4871	413534.62	1.18	ref	ref
Cancer	625	47918.86	1.30	1.09 (1.00-1.18)	1.36 (1.25–1.49)**
**Sex**					
**Female**					
Cancer free	8594	730867.44	1.18	ref	ref
Cancer	1727	118647.17	1.46	1.24 (1.18–1.31)**	1.59 (1.51–1.68)**
**Male**					
Cancer free	5493	689721.13	0.80	ref	ref
Cancer	886	84615.23	1.05	1.31 (1.22–1.41)**	1.70 (1.57–1.83)**
**CCI**					
**< 3**					
Cancer free	9104	818624.36	1.11	ref	ref
Cancer	878	43079.45	2.04	1.77 (1.65–1.90)**	1.92 (1.79–2.05)**
**≥ 3**					
Cancer free	4983	601964.21	0.83	ref	ref
Cancer	1735	160182.95	1.08	1.43 (1.35–1.51)**	1.46 (1.38–1.55)**
**Urbanization level**					
**Urban**					
Cancer free	5573	565360.05	0.99	ref	ref
Cancer	1067	84525.56	1.26	1.29 (1.20–1.37)**	1.62 (1.51–1.73)**
**Suburban**					
Cancer free	2983	307769.87	0.97	ref	ref
Cancer	519	41462.44	1.25	1.29 (1.18–1.42)**	1.62 (1.47–1.79)**
**Rural**					
Cancer free	5531	547458.65	1.01	ref	ref
Cancer	1027	77274.39	1.33	1.32 (1.24–1.41)**	1.66 (1.55–1.78)**

Incidence: incidence density (100 per person-years)

*: *p* <.05, **: *p* <.001, CHR: crude hazard ratio, AHR: adjusted hazard ratio controlling for age, sex, income, region, urbanization level, and CCI score, CCI: Charlson comorbidity index, CI: confidence interval, PY: person-years

The incidence densities of anxiety in patients with different cancer types are summarized in Table 6. Particularly high risks of anxiety were observed in patients with nose cancer (AHR: 3.12, 95% CI: 2.41–4.03), leukemia (AHR: 2.54, 95% CI: 1.63–3.96), thyroid cancer (AHR: 2.34, 95% CI: 1.84–2.97), and oral cancer (AHR: 2.04, 95% CI: 1.65–2.52).

**Table 6:** Risk of anxiety disorder by cancer type

Variables	Total	Anxiety				
	*N*	Cases	PY	Incidence	CHR (95% CI)	AHR (95% CI)
**Cancer type**						
Colon cancer	4754	404	31338.01	1.29	1.34 (1.20–1.50)**	1.72 (1.54–1.93)**
Lung cancer	2760	91	7486.20	1.22	1.13 (0.91–1.40)	1.49 (1.20–1.86)**
Breast cancer	4359	547	37342.59	1.46	1.26 (1.14–1.38)**	1.52 (1.37–1.68)**
Liver cancer	3909	187	13641.23	1.37	1.38 (1.18–1.61)**	1.88 (1.60–2.21)**
Oral cancer	1791	120	10108.06	1.19	1.69 (1.39–2.07)**	2.04 (1.65–2.52)**
Prostate cancer	1351	95	7707.67	1.23	1.45 (1.16–1.82)*	1.93 (1.62–2.46)**
Thyroid cancer	1107	137	11055.85	1.24	1.89 (1.55–2.31)**	2.34 (1.84–2.97)**
Skin cancer	452	42	3589.75	1.17	1.76 (1.23–2.51)*	1.92 (1.32–2.78)**
Stomach cancer	1591	101	7789.31	1.30	1.44 (1.17–1.79)**	1.87 (1.50–2.33)**
Gynecological cancer	2977	320	25083.23	1.28	0.98 (0.87–1.10)	1.21 (1.07–1.37)*
Brain cancer	507	29	3276.08	0.89	1.27 (0.85–1.89)	1.66 (1.08–2.55)*
Urological cancer	1887	137	12489.51	1.10	1.06 (0.88–1.27)	1.44 (1.19–1.74)**
Leukemia	455	31	2292.10	1.35	2.07 (1.39–3.08)*	2.54 (1.63–3.96)**
Bone cancer	106	10	717.35	1.39	1.63 (0.81–3.25)	2.00 (0.94–4.26)
Nose cancer	1186	105	8488.55	1.24	2.12 (1.70–2.65)**	3.12 (2.41–4.03)**

Incidence: incidence density (100 per person-years)

*: *p* <.05, **: *p* <.001, CHR: crude hazard ratio, AHR: adjusted hazard ratio controlling for age, sex, income, region, urbanization level, and CCI score, CCI: Charlson comorbidity index, CI: confidence interval, PY: person-years

## Discussion

Our findings reveal a significant bidirectional association between anxiety disorder and cancer. In our first analysis, we discovered that patients with anxiety were more likely to develop cancer than those without anxiety, with such patients having particularly high risks of developing thyroid, skin, prostate, nose, brain, urological, breast, and lung cancers. In our second analysis, we found that individuals with cancer had a greater likelihood of developing an anxiety disorder compared to those without cancer. The risk of developing an anxiety disorder was notably higher for all types of cancer included, except for bone cancer. For bone cancer, although the risk estimate was high, it was not statistically significant, with a wider 95% confidence interval (AHR = 2.00, 95% CI: 0.94–4.26), suggesting low statistical power.

### Association between anxiety disorder and the risk of developing cancer

Our study found that individuals with anxiety have a significantly higher risk of developing cancer (CHR = 1.21, AHR = 1.29) compared to those without anxiety, slightly exceeding results from previous study [32]. One study found that anxiety and depression increase cancer risk and mortality by 41%, and anxiety-related insomnia treatments like sedative-hypnotics may further elevate cancer risk [33, 34].

Over a 14-year follow-up, cancer incidence was higher in those with anxiety (1.08 vs. 0.90 per 100 person-years), consistent with another study reporting a rate of 1.14 in individuals with generalized anxiety disorder [16]. Research has shown that lifestyle factors such as poor diet, lack of physical activity, smoking, and alcohol use, along with biological factors like viral oncogenes and impaired DNA repair, may increase cancer risk in those with anxiety [25, 35, 36].

Gender and age also play significant roles. Anxiety disorders are more prevalent in women, and sex-specific differences in brain function could influence cancer risk [37]. While men with anxiety often engage in riskier behaviors like smoking and drinking, increasing their cancer risk, women may seek timely treatment, potentially lowering their risk [38]. Older adults are more likely to develop cancer, and when combined with anxiety, this risk may be heightened [39].

We examined 15 cancer types and found a high risk of thyroid, skin, prostate, nose, brain, urological, breast, and lung cancers in individuals with anxiety, with thyroid cancer showing a particularly high risk. This aligns with studies linking stress and thyroid cancer, as anxiety may affect thyroid function, creating an environment conducive to cancer development [40–43].

Our findings also revealed an association between anxiety and skin cancer, adding to evidence that psychological factors may impact skin health and cancer risk [44]. Anxiety may weaken the skin’s defense against ultraviolet rays, increasing vulnerability [45]. Anxiety has also been linked to exacerbating preexisting skin conditions, which may contribute to a higher cancer risk [45].

Finally, we observed a heightened risk of prostate cancer in individuals with anxiety, consistent with previous studies [32]. Another study also reported elevated levels of anxiety in men actively seeking prostate cancer screening [46]. These results underscore the need for interventions addressing anxiety disorders, which could potentially reduce cancer risk. Despite the varying results, we can infer that those with anxiety disorders have an increased likelihood of developing cancer. Interventions focusing on anxiety disorders could thus assist in cancer prevention.

### Association between cancer and the risk of developing anxiety disorder

Our results demonstrate that individuals with cancer are at a significantly higher risk of developing anxiety disorders compared to those without cancer. The incidence of anxiety was also higher among those with cancer, consistent with previous studies reporting that around 7.6% of cancer patients experience anxiety, with generalized anxiety disorder (GAD) being the most prevalent form [22, 47–49]. Most individuals with cancer experience anxiety upon receiving their diagnosis and as the cancer progresses [22, 48]. Anxiety may also be experienced during cancer treatment or screening or be triggered by anticipation of a recurrence [48], highlighting the need for anxiety assessments throughout cancer care.

Furthermore, we found that the risk of anxiety increased for nearly all cancer types, except bone cancer, with the highest risk observed in patients with nose cancer. This may be due to the facial disfigurement and social isolation that often accompany nose cancer. Patients may also experience anosmia (loss of smell), which can further heighten anxiety [50–52].

Leukemia patients had the second-highest risk of anxiety. Treatments like chemotherapy and bone marrow transplants, combined with the uncertainty of relapse, can trigger significant psychological distress [53]. The therapeutic regimen for managing chronic lymphocytic leukemia does not involve maintenance therapy after remission occurs; thus, patients with this condition often experience feelings of anxiety and apprehension regarding the possibility of relapse [54].

Similarly, thyroid cancer patients also showed elevated anxiety risk, possibly due to the thyroid’s role in mood regulation and the appearance-related concerns following surgery [55]. A study reported a significant link between perceived neck appearance and anxiety among patients with cancer who underwent a thyroidectomy [56]. Although some forms of thyroid cancer have a favorable overall prognosis, the condition can still prompt anxiety. Proper management and support are crucial to ensure patients successfully navigate this experience and maintain high quality of life.

Finally, in our study, the risk of anxiety was high in individuals with oral cancer. our results align with a study that revealed that participants experienced considerable anxiety regarding tumors related to human papillomavirus (HPV) infection upon receiving their initial diagnosis of HPV-related oropharyngeal cancer [57]. Oral cancer differs considerably from other forms of cancer. For example, treatment of advanced oral cancer often involves invasive procedures that can have a lasting impact on a patient’s ability to eat or speak as well as on their confidence in their appearance. These factors emphasize the need for holistic care that addresses both the physical and psychological burdens of cancer.

### Strengths and limitations

This is the first study to evaluate the bidirectional relationship between cancer and anxiety in the Taiwanese population, using a large, population-based dataset and analyzing multiple cancer types. However, several limitations warrant caution in interpreting the results. First, we did not exclude patients with multiple diagnoses, as anxiety disorders often co-occur with other mental conditions [58], we did not exclude patients with multiple diagnoses; a study highlighted that a solitary diagnosis of an anxiety disorder cannot be universally applied to real-world scenarios in which multiple diagnoses often co-occur [59]. We only included patients with anxiety disorder-related principal diagnoses and at least two clinical visits for greater precision. Second, the retrospective design limits claims of causality. Third, we did not account for confounding factors like environmental exposures or health behaviors, which may have introduced bias. Fourth, we did not assess cancer risk for specific anxiety disorders. Fifth, as our analysis relied on Cox regression, we did not test for the proportional hazards assumption, and violations of this assumption could influence the results, particularly over long follow-up periods. Sixth, we did not account for competing risks, such as death from causes unrelated to the outcomes of interest. Lastly, the findings may not be generalizable beyond the Taiwanese population. Future research should address these limitations by excluding multiple anxiety diagnoses, adding more confounders, using a prospective design, testing the proportional hazards assumption for robustness, and incorporating competing risks in the analysis.

## Conclusions

Our findings show a bidirectional link between cancer and anxiety disorder. Anxiety patients had a higher risk of developing cancers, especially thyroid cancer, while cancer patients, particularly with nose cancer, were more likely to develop anxiety. This emphasizes the importance of regular anxiety assessments for cancer patients to better address their mental health needs. However, several limitations must be considered in our study. The retrospective design and multiple diagnoses, potential unaccounted confounding variables, and focus on a Taiwanese population may limit generalizability. Given cancer’s complexity and the psychological burden it imposes, these insights can guide healthcare providers in developing more holistic treatment strategies that address both physical and mental health aspects. Future studies should adopt a prospective approach to deepen understanding.

## Data Availability

The data employed in this study were obtained from the Taiwan National Health Insurance Research Database; formal data requests should be directed to the Health and Welfare Data Science Center, Ministry of Health and Welfare (HWDC, MOHW).

## Abbreviations

COVID-19: Coronavirus disease 2019
NHIRD: National Health Insurance Research Database
HWDC: Health and Welfare Data Science Center
MOHW: Ministry of Health and Welfare
HPV: Human Papilloma Virus
